# Blockade of cannabinoid 1 receptor improves glucose responsiveness in pancreatic beta cells

**DOI:** 10.1111/jcmm.13523

**Published:** 2018-02-12

**Authors:** Hanho Shin, Ji Hye Han, Juhwan Yoon, Hyo Jung Sim, Tae Joo Park, Siyoung Yang, Eun Kyung Lee, Rohit N. Kulkarni, Josephine M. Egan, Wook Kim

**Affiliations:** ^1^ Department of Molecular Science and Technology Ajou University Suwon South Korea; ^2^ School of Life Science Ulsan National Institute of Science and Technology (UNIST) Ulsan South Korea; ^3^ Center for Genomic Integrity Institute for Basic Science Ulsan South Korea; ^4^ Department of Pharmacology Ajou University School of Medicine Suwon South Korea; ^5^ Department of Biomedical Sciences Ajou University Graduate School of Medicine Suwon South Korea; ^6^ Department of Biochemistry College of Medicine The Catholic University of Korea Seoul South Korea; ^7^ Department of Islet Cell and Regenerative Biology Joslin Diabetes Center and Department of Medicine Harvard Medical School Harvard Stem Cell Institute Boston MA USA; ^8^ Laboratory of Clinical Investigation National Institute on Aging National Institutes of Health Baltimore MD USA

**Keywords:** β‐cell function, cannabinoid 1 receptor, glucokinase, glucose transporter 2, insulin secretion

## Abstract

Cannabinoid 1 receptors (CB1Rs) are expressed in peripheral tissues, including islets of Langerhans, where their function(s) is under scrutiny. Using mouse β‐cell lines, human islets and CB1R‐null (*CB1R* ^−/−^) mice, we have now investigated the role of CB1Rs in modulating β‐cell function and glucose responsiveness. Synthetic CB1R agonists diminished GLP‐1‐mediated cAMP accumulation and insulin secretion as well as glucose‐stimulated insulin secretion in mouse β‐cell lines and human islets. In addition, silencing CB1R in mouse β cells resulted in an increased expression of pro‐insulin, glucokinase (GCK) and glucose transporter 2 (GLUT2), but this increase was lost in β cells lacking insulin receptor. Furthermore, *CB1R* ^−/−^ mice had increased pro‐insulin, GCK and GLUT2 expression in β cells. Our results suggest that CB1R signalling in pancreatic islets may be harnessed to improve β‐cell glucose responsiveness and preserve their function. Thus, our findings further support that blocking peripheral CB1Rs would be beneficial to β‐cell function in type 2 diabetes.

## 1 INTRODUCTION

Insulin‐producing pancreatic β cells have to secrete insulin in a way that maintains the blood glucose level in a narrow range at all times. Glucose is the primary stimulus for insulin secretion, a process that requires glucose sensing.^1^ Pancreatic β cells can sense and respond to changing blood glucose concentrations with the help of glucose transporter 2 (GLUT2) and glucokinase (GCK), which are the glucose‐sensing machinery.^1^ Blood glucose is transported into the β cells through GLUT2, and GCK traps glucose in the cytoplasm by immediate phosphorylation. Glucose metabolism in β cells generates ATP that inhibits potassium efflux through K_ATP_ channels, resulting in β‐cell membrane depolarization and entry of extracellular Ca^2+^ through voltage‐dependent Ca^2+^ channels, which then initiate insulin release. The glucose dependency of insulin secretion is greatly enhanced by increased adenylyl cyclase (AC) activity and in the fed state, enhanced AC activity is generally controlled by the incretins, glucagon‐like peptide‐1 (GLP‐1) and glucose‐dependent insulinotropic peptide (GIP). The binding of GLP‐1 to its specific receptors (GLP‐1Rs) on β cells results in activation of AC, cAMP production and subsequent activation of PKA and the Epac family. This sequence of events leads to enhanced glucose‐dependent exocytosis of insulin from insulin‐secretory vesicles 2, 3 and other downstream effects such as improved insulin biosynthesis and increased pro‐insulin, GLUT2 and GCK expressions.^4^, ^5^, ^6^ Accordingly, altered glucose‐sensing machinery of β cells leads to defects in glucose‐stimulated insulin secretion (GSIS). Indeed, islets from diabetic Zucker rats have impaired GSIS due to defective glucose sensing associated with a reduction in the expressions of GLUT2 and GCK.^7^ Moreover, β‐cell–specific knockout of GLUT2 or GCK led to severe hyperglycaemia and infant death due to impaired GSIS and, conversely, re‐expression of GLUT2 in β cells rescued GLUT2‐null mice from infant death and restored normal GSIS.^8^, ^9^

β cells synthesize and secrete insulin as well as the known endogenous cannabinoids (ECs).^10^, ^11^, ^12^ The ECs, 2‐arachidonoylglycerol (2‐AG) and anandamide (AEA), are lipid transmitters that are not pre‐stored in granules, but are synthesized and released from cells only “on demand” by Ca^2+^‐dependent enzymes in the brain, fat, macrophages, and liver, in addition to islets, following membrane depolarization.^10^, ^11^, ^12^, ^13^, ^14^, ^15^ Thus, both insulin secretion and EC synthesis are controlled by cell depolarization and Ca^2+^ mobilization. The biological effects of ECs are mediated by two G protein‐coupled receptors (CB1R and CB2R), which, when activated, inhibit AC activity and cAMP accumulation.^15^, ^16^ CB1Rs are expressed in neurons and peripheral tissues such as liver, adipose tissue and skeletal muscle and contribute to food intake and peripheral metabolic regulation including lipid and glucose homoeostasis and insulin sensitivity.^17^, ^18^, ^19^ In fact, as ECs and CB1Rs are overactive in obesity and type 2 diabetes (T2D), many scientists have been interested in blockade of CB1R as a new therapeutic approach for the treatment of obesity‐related diseases. In addition, the majority of reports, including our previous study,^20^, ^21^, ^22^ found that rodent and human β cells express CB1Rs, whereas CB2Rs were not expressed in β cells^11^, ^20^, ^23^, ^24^; however, there are also reports to the contrary.^10^, ^11^, ^12^, ^23^, ^25^

EC treatment of isolated mouse islets was reported to inhibit GSIS and lower intracellular Ca^2+^ levels^24^; there are similar findings from another group.^25^, ^26^ However, the published literature is conflicting as regards the coupling of CB1Rs with Gαi or Gαs as well as their effects on insulin secretion. It has been reported that in certain circumstances, CB1R stimulation leads to Gαs coupling, AC activation and increased GSIS.^11^, ^16^, ^27^, ^28^, ^29^, ^30^, ^31^ In addition, there are reports of EC treatment of mouse islets leading to decrease in cAMP,^16^, ^28^ while increasing intracellular calcium and insulin secretion.^16^ Here, we evaluated human islets, several β‐cell lines and CB1R‐null (*CB1R* ^−/−^) mice in order to outline what the relevance of β‐cell–derived ECs might be to AC activity and insulin secretion. Moreover, we also investigated the effect of CB1Rs on the expression of pro‐insulin, GLUT2 and GCK.

## 2 MATERIALS AND METHODS

### 2.1 Materials and reagents

The Rat/Mouse Insulin ELISA Kit was from Linco Research, and the cAMP EIA kit was from Assay Designs. WIN55,212‐2, CP 55,940, arachidonyl‐2′‐chloroethylamide (ACEA) and AM251 were obtained from Cayman Chemical. Exendin‐4 (Ex‐4) was obtained from Bachem. The human CB1R cDNA was amplified by RT‐PCR from human pancreatic RNA (Stratagene), with oligo‐dT (18 bp) for the reverse transcription. The CB1R cDNA was incorporated into a mCerulean‐N1 vector for CB1R‐cerulean with the cerulean epitope.

### 2.2 Cell culture and insulin secretion and cAMP assays from cell lines

CHO‐K1, CHO‐GLP‐1R (CHO‐K1 cells stably transfected with GLP‐1R),^32^ CHO‐GLP‐1R‐CB1R (CHO‐GLP‐1R cells stably transfected with CB1R) 21 were maintained in DMEM/F‐12 medium with 10% FBS. βIRWT and βIRKO cells were established from control and β‐cell–specific insulin receptor (IR) knockout mice, respectively.^20^, ^22^, ^33^ Insulin‐secreting mouse β‐cell lines (MIN6 and βTC6 cells) were maintained in DMEM medium with 10% FBS (Invitrogen). For insulin secretion and cAMP assays, cells were plated in 12‐well plates, 1 and 3 days before transfection, respectively. Cells were washed three times in PBS and pre‐incubated for 2 hours in Krebs buffer containing 4 mmol/L glucose, at 37°C. Subsequently, the cells were treated with CB1R agonists (WIN 55,212‐2, CP 55,940, ACEA or 2‐AG) in the absence and presence of CB1R antagonist AM215 (2.5 μmol/L) for 15 minutes prior to stimulation with glucose (4, 15, or 25 mmol/L), forskolin (10 μmol/L) or Ex‐4 (10 or 25 nmol/L) for 20 minutes. At the end of the experiment, the buffer was collected, centrifuged to remove cellular debris and saved for quantification of insulin. The cells were lysed with 0.1 mol/L HCl and were centrifuged to remove cellular debris. The supernatant was collected for determination of cAMP levels and protein concentrations. The cAMP levels were measured using a cAMP EIA kit according to the manufacturer's instructions. The data were normalized to insulin content or protein concentration and estimated from three independent experiments, each performed in at least triplicate. Transfections of the expression vectors and siRNA (Santa Cruz) for CB1R were carried out 24 and 48 hours before adding CB1R agonist using Lipofectamine 2000 and RNAiMAX (Invitrogen), respectively. Scramble siRNA (Silencer Negative Control #1; Ambion) or empty vector was transfected for negative control.

### 2.3 Mice

*CB1R* ^−/−^ mice and their wild‐type littermates were developed and backcrossed to a C57Bl/6J background as previously described.^34^ Two‐ to three‐month‐old male *CB1R* ^+/+^ and *CB1R* ^−/−^ mice were used for this study. All animal care and experimental procedures followed National Institutes of Health guidelines and were approved by the National Institute on Aging Animal Care and Use Committee.

### 2.4 Islet isolation and measurement of insulin content and secretion in islets

Freshly isolated islets of Langerhans from human cadaveric donors were obtained from the Islet Cell Resource Center. Mouse islets were isolated from *CB1R* ^−/−^ and age‐matched *CB1R* ^+/+^ mice using collagenase digestion as previously described.^35^ For insulin secretion assays, we picked 10 size‐matched islets per tube in Krebs buffer containing 4 mmol/L glucose followed by 30‐minutes incubation at 37°C. We pelleted the islets and replaced the buffer with Krebs solution containing the indicated glucose concentration. After 10‐minutes incubation at 37°C, we collected the supernatant for insulin measurement and measured insulin content or protein concentration for normalization. Insulin levels were determined from three independent experiments performed in triplicate. Intra‐islet insulin content was measured using ice‐cold acid‐alcohol as we previously described.^6^

### 2.5 Immunostaining

For frozen sections, mice were anaesthetized, and pancreata were rapidly dissected, fixed in 4% paraformaldehyde, immersed in 20% sucrose before freezing and then sectioned at a thickness of 7 μm. After antigen unmasking, the slides were blocked with 5% BSA/PBS and incubated at 4°C, with guinea pig anti‐insulin (1:500; Millipore), rabbit anti‐GCK (1:100; Santa Cruz) and rabbit anti‐GLUT2 (1:200; Santa Cruz), followed by secondary antibodies (Invitrogen). Slides were viewed under an LSM‐710 confocal microscope (Carl Zeiss). Using LSM Image Browser software (Carl Zeiss), multiple sections from three mice per genotype, separated by at least 200 μm from each section, were assessed for quantification of GCK and GLUT2.

### 2.6 Quantitative real‐time PCR analysis

Total RNAs were isolated from frozen mouse islets or βTC6 cells using the TRIzol reagent (Thermo Fisher Scientific, Waltham, MA, USA), according to the manufacturer's protocol. All extractions were followed by DNase I treatment (Invitrogen). After reverse transcription (RT) using random hexamers and reverse transcriptase (Toyobo, Osaka, Japan), the mRNA abundance was assessed using a CFX Connect^TM^ Real‐Time PCR Detection System (Bio‐Rad, Hercules, CA, USA) and the SYBR green PCR master mix (Kapa Biosystems, Wilmington, MA, USA). The *18S* gene was used for normalization to quantify the relative mRNA expression levels. Gene‐specific primer sequences were summarized in Table S1.

### 2.7 Immunoblotting

Protein samples were extracted from cells using RIPA buffer (50 mmol/L Tris‐HCl at pH 7.4, 150 mmol/L NaCl, 1% NP‐40, 0.1% SDS, 1 mmol/L EDTA) containing protease and phosphatase inhibitor cocktails (Calbiochem). They were then subjected to Tris‐Glycine PAGE (Invitrogen), immunoblotted with rabbit anti‐CB1R (1:500; Frontier Science), anti‐insulin (1:1000; Santa Cruz), anti‐GCK (1:500; Abcam), anti‐GLUT2 (1:1000; Santa Cruz) or mouse anti‐β‐actin (1:10000; Abcam), and visualized by ECL (GE Health). Whole pancreata were homogenized by a motor‐driven pestle in ice‐cold RIPA buffer containing protease and phosphatase inhibitor cocktails. Homogenates were solubilized by end‐over‐end mixing at 4°C for 60 minutes and subjected to centrifugation. Total protein was determined using Bradford assay (Bio‐Rad).

### 2.8 Statistical analysis

Quantitative data were presented as the mean ± SEM. Differences between mean values for variables within individual experiments were compared statistically by Student's *t* test. Comparisons were performed using GraphPad Prism (GraphPad Software). A *P* value of <.05 was considered statistically significant.

## 3 RESULTS

### 3.1 Activation of CB1Rs decreases cAMP accumulation

**Figure 1:**
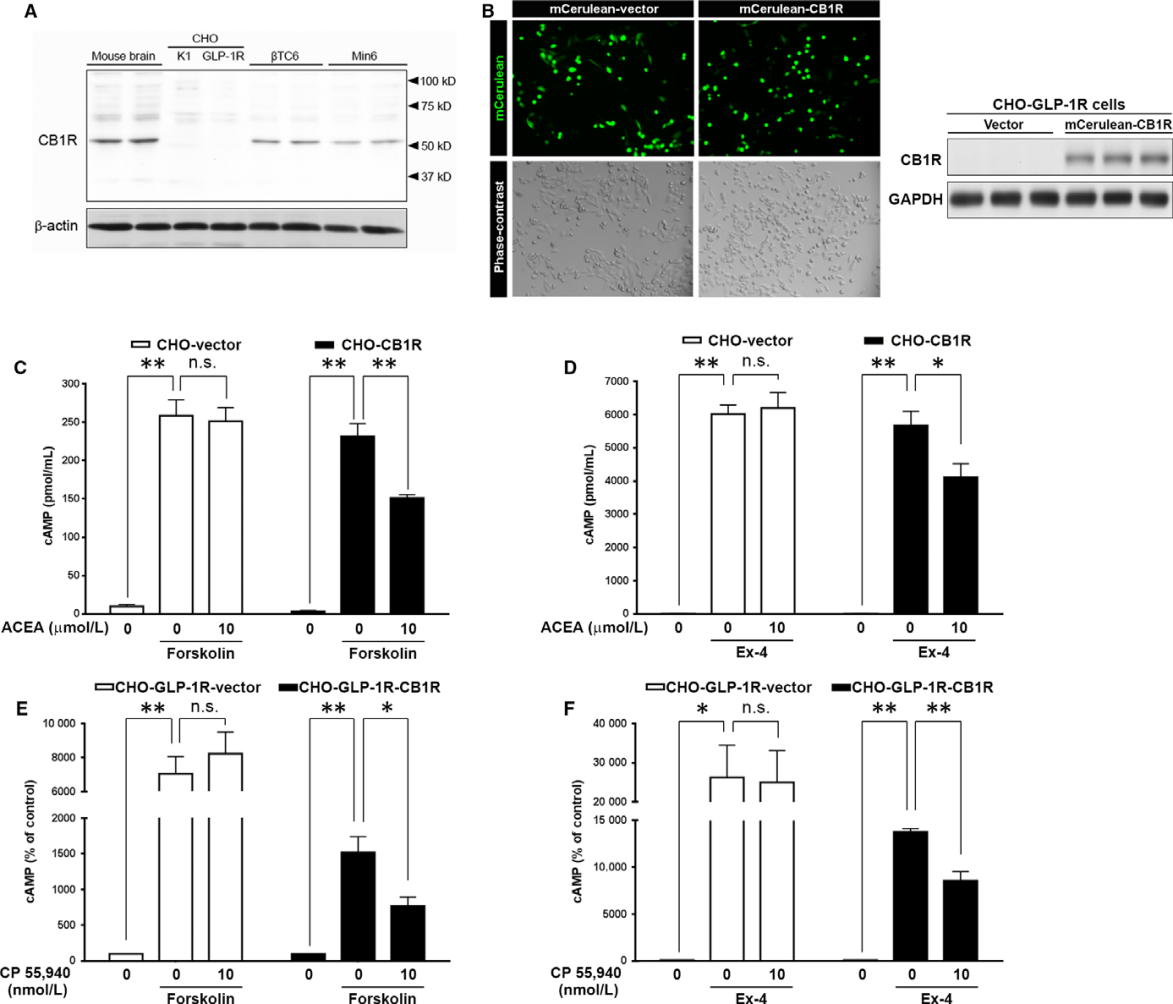
Effects of CB1R agonists on intracellular cAMP accumulation in CHO‐GLP‐1R cells. A, Western blot analysis showing CHO‐K1 and CHO‐GLP‐1R (CHO‐K1 cells stably transfected with rat GLP‐1R) do not express CB1Rs, unlike insulin‐secreting mouse β‐cell lines (βTC6 and MIN6). Mouse brain was used as a positive control, and β‐actin was used as a loading control. B, Overexpression of CB1R in CHO‐K1 cells. Representative images of the mCerulean‐vector‐ and mCerulean‐CB1R‐transfected CHO‐K1 cells under a fluorescence microscope 24 h after transfection. Western blot analysis of CB1R expression in CHO‐K1 cells 24 h after mCerulean‐CB1R transfection is shown on the right. C, Effects of CB1R overexpression on forskolin‐mediated cAMP accumulation. CHO‐K1 cells stably expressing GLP‐1R were transiently transfected with empty vector (CHO‐vector) or CB1R (CHO‐CB1R) and pre‐incubated with ACEA for 15 min prior to stimulation with forskolin. D, Effects of CB1R overexpression on Ex‐4‐mediated cAMP accumulation in CHO‐vector and CHO‐CB1R cells. The cells were pre‐treated with ACEA for 15 min before the subsequent addition of Ex‐4 for an additional 20 min. E, Forskolin‐mediated cAMP accumulation in CHO‐GLP‐1R cells stably transfected with empty vector (CHO‐GLP‐1R‐vector) or CB1R (CHO‐GLP‐1R‐CB1R). The cells were pre‐incubated with CP 55,940 for 15 min prior to stimulation with forskolin. F, Ex‐4‐mediated cAMP accumulation in CHO‐GLP‐1R‐vector and CHO‐GLP‐1R‐CB1R cells. The cells were pre‐treated with CP 55,940 for 15 min prior to stimulation with Ex‐4. All values were normalized to protein concentration. Data are shown as the mean ± SEM from at least 3 independent experiments. *P < .05; **P < .01, n.s. = not significant

Despite conflicting studies,^36^, ^37^ the majority of reports on the subject suggest that CB1Rs are present in pancreatic β cells.^10^, ^11^, ^15^, ^23^, ^24^, ^38^, ^39^ Western blot analysis confirmed that CB1Rs are expressed in mouse insulinoma cell lines (βTC6 and MIN6 cells) but are lacking in CHO cell lines (CHO‐K1 and CHO‐GLP‐1R) stably transfected with vector and GLP‐1R 32 (Figure 1A).

As activation of CB1R leads to reduced intracellular cAMP by inhibiting AC via Gαi class of heterotrimeric G proteins,^15^, ^16^ we surmised that ECs upon their discharge from β cells, which occurs after membrane depolarization,^10^, ^11^, ^12^, ^15^ would influence cAMP accumulation and insulin secretion. To prove whether ECs inhibit AC activity by activation of CB1Rs, we first transiently transfected CHO‐GLP‐1R cells with vector (CHO‐vector) or CB1R (CHO‐CB1R) and activated AC in these cells using forskolin (Figure 1B). As expected, this led to increased intracellular cAMP levels in CHO‐vector cells, whereas ACEA, a highly selective CB1R agonist, did not impact the effect of forskolin (Figure 1C). However, in CHO‐CB1R cells, ACEA significantly reduced forskolin‐mediated intracellular cAMP generation (Figure 1C). Next, we investigated the effects of ACEA on cAMP accumulation caused by Exendin‐4 (Ex‐4, a potent ligand of the GLP‐1R) in CHO‐vector and CHO‐CB1R cells. Under the conditions of high GLP‐1R expression, Ex‐4 is a powerful stimulus to AC activation and intracellular cAMP accumulation (Figure 1D). The inhibitory effects of ACEA on Ex‐4‐stimulated cAMP accumulation were not observed in CHO‐vector cells, which is likely due to lack of CB1Rs (Figure 1A). However, ACEA treatment in CHO‐CB1R cells transiently transfected with CB1R led to reduced cAMP accumulation (Figure 1D). To further confirm the effects of CB1R agonism on cAMP accumulation, we used another CB1R agonist CP 55 940 and alternate CHO‐GLP‐1R cells stably transfected with empty vector (CHO‐GLP‐1R‐vector) or CB1R (CHO‐GLP‐1R‐CB1R).^21^ Consistently, CP 55 940 abrogated both forskolin‐ and Ex‐4‐mediated cAMP accumulation in CHO‐GLP‐1R‐CB1R cells, but not in CHO‐GLP‐1R‐vector (Figure 1E, F), confirming the inhibitory action of ECs on AC activity in a receptor‐dependent manner.

### 3.2 Activation of CB1Rs decreases insulin secretion from pancreatic β cells

**Figure 2:**
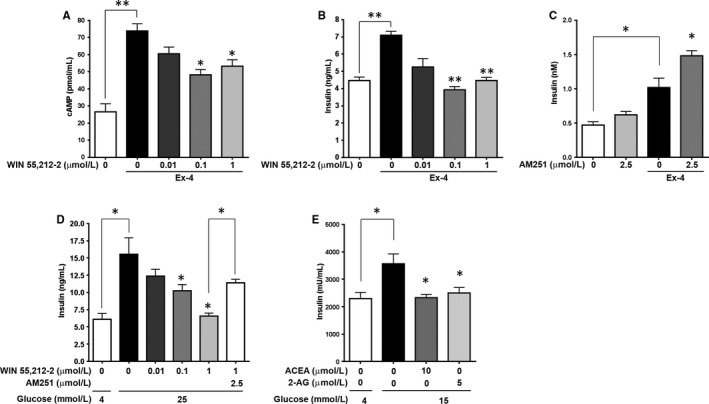
Effects of CB1R agonists on insulin secretion. A and B, Relative intracellular cAMP (A) and insulin (B) concentrations secreted from βTC6 cells treated with the synthetic CB1R agonist WIN55,212‐2 before the subsequent addition of Ex‐4. C, Effects of blocking CB1R on Ex‐4‐mediated insulin secretion in human islets. Human islets were pre‐treated with a CB1R inverse agonist AM251 before the subsequent addition of Ex‐4. D, Relative insulin concentrations secreted from βTC6 cells in response to glucose in the absence or presence of WIN55,212‐2, AM251 or both. E, Relative insulin concentrations secreted from human islets in response to glucose in the absence or presence of ACEA or 2‐AG. All values were normalized to protein concentration. Data are shown as the mean ± SEM from three independent experiments. *P < .05; **P < .01

Having shown that ECs inhibit forskolin‐ and Ex‐4‐mediated AC activation and cAMP accumulation, we subsequently investigated their effects in β cells. Ex‐4‐mediated cAMP accumulation in βTC6 cells was significantly decreased by another synthetic CB1R agonist WIN55,212‐2 in a concentration‐dependent manner (Figure 2A). Consistently, WIN55,212‐2 also abrogated Ex‐4‐mediated insulin secretion from βTC6 cells (Figure 2B). Using human islets, we further confirmed that the insulinotropic effects of Ex‐4 were enhanced by blocking CB1Rs with AM251, a CB1R antagonist (Figure 2C). These results prove that ECs antagonize incretin‐mediated effects in β cells.

We then examined the effect of CB1R on GSIS. As expected, pharmacological activation of CB1R by WIN55,212‐2 inhibited GSIS from βTC6 cells in a concentration‐dependent manner, and this was counteracted by pre‐incubation of the cells with AM251 (Figure 2D). Furthermore, ACEA and 2‐AG inhibited GSIS from human islets (Figure 2E). Overall, these results favour the conclusion that ECs are inhibitory mediators of AC activity and insulin secretion in β cells.

### 3.3 Effects of CB1R ablation on gene expression

**Figure 3:**
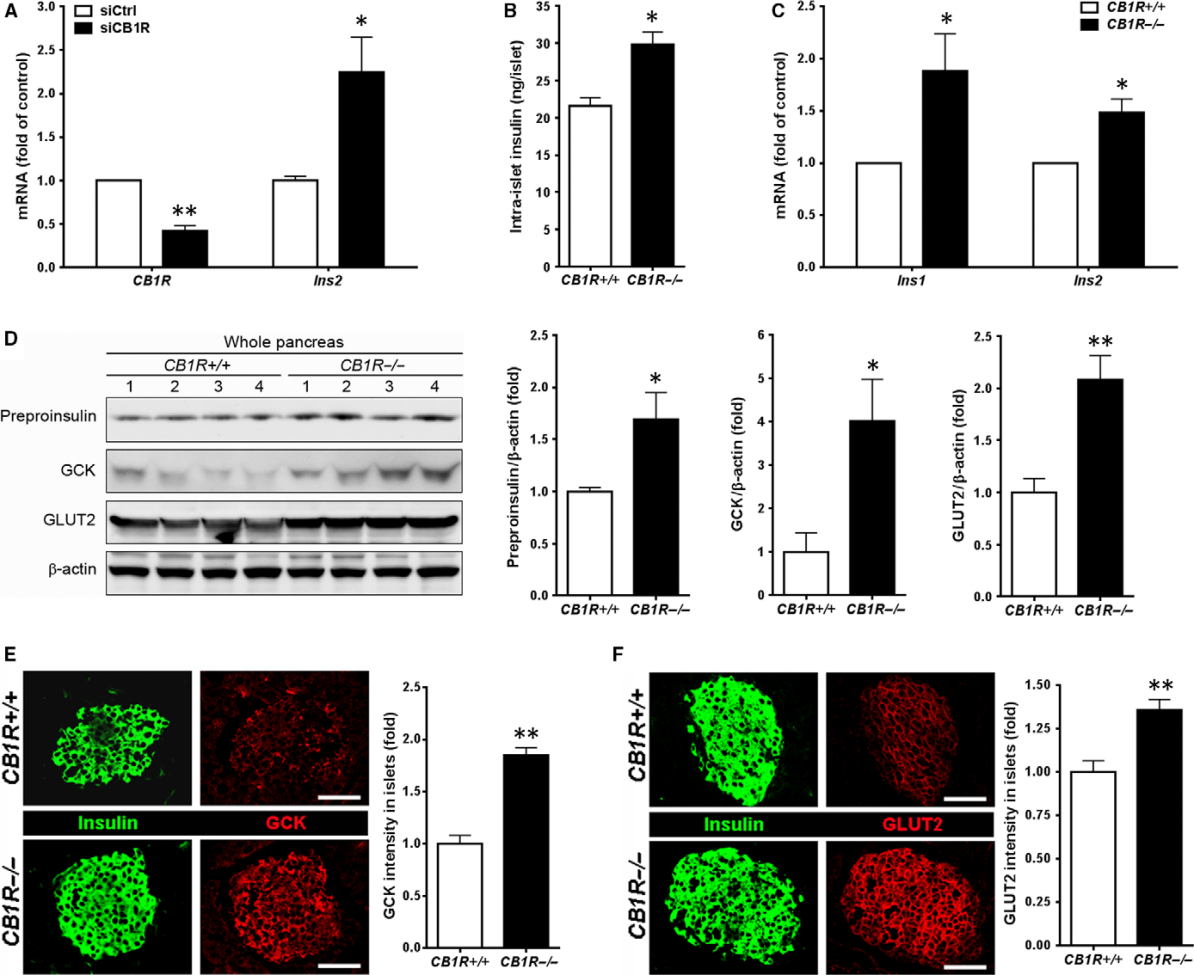
Effects of blocking CB1R on intra‐β‐cell insulin content and glucokinase (GCK) and glucose transporter 2 (GLUT2) expressions. A, *CB1R* and *Insulin* (*Ins2*) mRNA levels in βTC6 cells transfected with control (siCtrl) or CB1R (siCB1R) siRNA. B, Intra‐islet insulin content in islets isolated from *CB1R* ^+/+^ and *CB1R* ^−/−^ mice. Insulin was extracted from islets freshly isolated from *CB1R* ^+/+^ and *CB1R* ^−/−^ mice using acid‐alcohol (n = 3 separate isolates). Size‐matched 10 islets per tube were analysed, and data were normalized to protein concentration. C, *Insulin* (*Ins1* and *Ins2*) mRNA levels in islets isolated from *CB1R* ^+/+^ and *CB1R* ^−/−^ mice (n = 4 separate isolates). Data were normalized to *18S* ribosomal RNA levels. D, Western blot analysis of preproinsulin, GCK and GLUT2 expressions in total lysates prepared from whole pancreata of overnight‐fasted *CB1R* ^+/+^ and *CB1R* ^−/−^ (n = 4 per genotype) mice. Signals on Western blots were quantified by densitometry and are shown on the right. E and F, Immunofluorescent staining for GCK (E) and GLUT2 (F) in islets of overnight‐fasted *CB1R* ^+/+^ and *CB1R* ^−/−^ mice. Scale bar, 50 μm. Relative signal intensity for the indicated proteins in islets is shown on the right (n = 3 per genotype). Quantification of GCK and GLUT2 intensities was shown on the right. Data are shown as the mean ± SEM from three independent experiments. *P < .05; **P < .01

Next, we measured the abundance of intra‐islet insulin and insulin mRNA in β cells. Knockdown of CB1Rs by siRNA in βTC6 cells enhanced insulin gene expression (Figure 3A). Freshly isolated islets from *CB1R* ^−/−^ mice had increased insulin content after acid‐alcohol extraction compared to those of age‐matched wild‐type (*CB1R* ^+/+^) littermates (Figure 3B), most likely as a direct result of up‐regulation of insulin gene expression (Figure 3C). Consistently, Western blot analysis confirmed that pancreas of *CB1R* ^−/−^ mice had significantly increased level of pro‐insulin compared to those of age‐matched *CB1R* ^+/+^ mice (Figure 3D). In addition, we measured protein levels of glucose‐sensing apparatus involved in the early steps of GSIS and glucose metabolism in β cells. Western blot analysis showed that pancreas of *CB1R* ^−/−^ mice had significantly increased level of GCK and GLUT2 compared to those of *CB1R* ^+/+^ mice (Figure 3D). Consistently, levels of GCK (Figure 3E) and GLUT2 (Figure 3F) in fasting state were significantly higher in β cells of *CB1R* ^−/−^ mice compared to those of *CB1R* ^+/+^ mice.

### 3.4 Inhibitory effects of CB1R on gene expression depend on insulin receptor (IR) signalling

**Figure 4:**
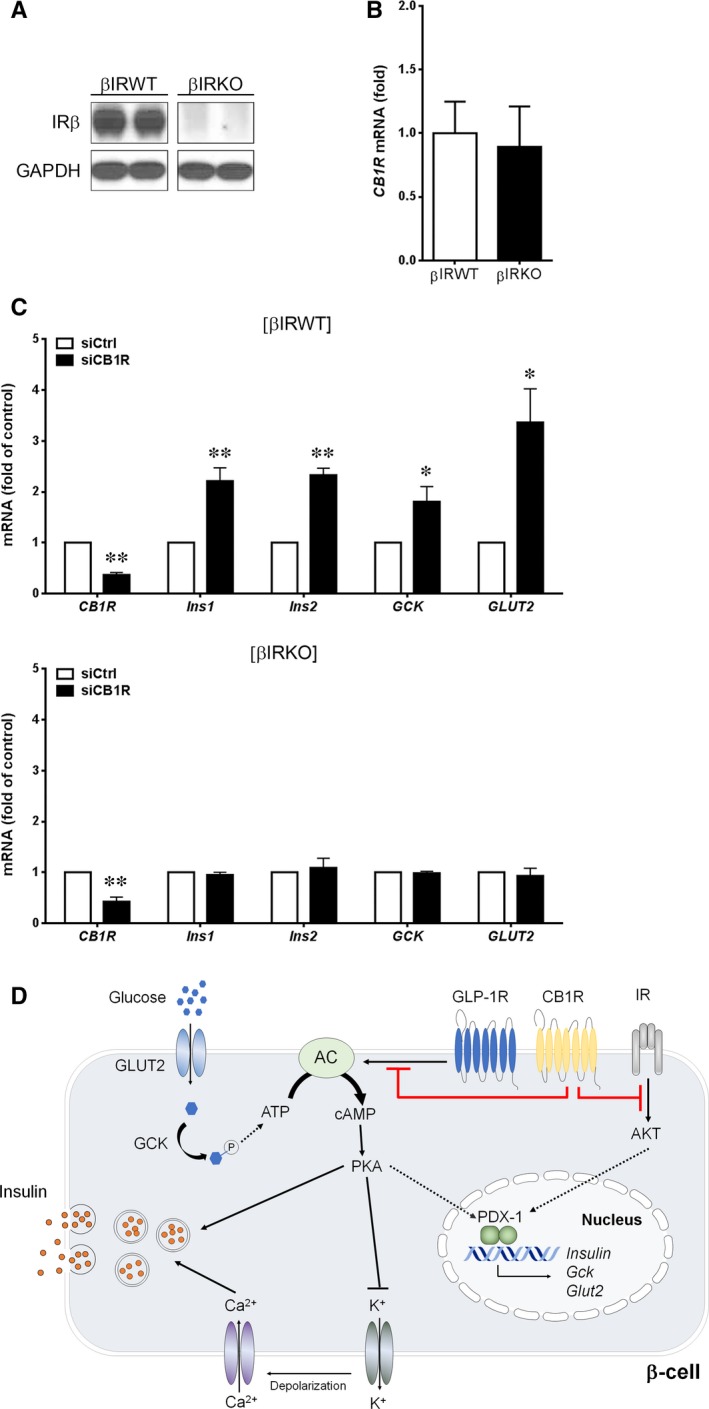
Effects of CB1R on gene expression depends on insulin receptor (IR). A, Western blot analysis of IR β‐subunit (IRβ) and GAPDH in βIRWT and βIRKO cells. B, Quantitative real‐time PCR analysis of CB1R expression in βIRWT and βIRKO cells. Data were normalized to *18S* ribosomal RNA levels. C, Quantitative real‐time PCR analysis of CB1R, insulin, GCK and GLUT2 expression in βIRWT and βIRKO cells transfected with control (siCtrl) or CB1R (siCB1R) siRNA. D, Schematic unifying the regulation of β‐cell function by ECs and CB1Rs. Data are shown as the mean ± SEM from three independent experiments. *P < .05; **P < .01

Our previous reports have shown direct evidence of physical and functional interactions between CB1R and insulin receptors (IRs) in β cells, in which activation of CB1R suppressed IR signalling pathway via IRS2‐AKT‐FoxO1 by diminishing IR kinase activity.^20^, ^22^ As IR signalling is a key regulator that promotes the expression of insulin, GCK and GLUT2 in β cells, we investigated whether CB1Rs regulate the expression of insulin, GCK and GLUT2 in IR‐dependent manner. For this, we employed β cells established from wild‐type (βIRWT) and β‐cell–specific IR knockout (βIRKO) mice 20, 22, 33 (Figure 4A), in which expression levels of CB1Rs are similar (Figure 4B). Knockdown of CB1Rs by siRNA in βIRWT cells enhanced the expression of insulin, GCK and GLUT2, whereas knockout of IR abolished the ability of CB1R to regulate the expression of insulin, GCK and GLUT2 (Figure 4C), suggesting that CB1R regulates the expression of insulin, GCK and GLUT2 by inhibiting IR signalling (Figure 4D).

## 4 DISCUSSION

As mentioned in the introduction, there are many reports of CB1R expression on pancreatic β cells in mouse and human 10, 11, 16, 23, 24, 38, 39 and we concur.^20^, ^21^, ^22^, ^23^ Recent reports, including our own,^20^ have also found that β cells contain the other components of EC system including the necessary enzymes for their biosynthesis and degradation, and have the capacity to generate ECs in response to glucose stimulation even when islets are isolated from the pancreas.^10^, ^11^, ^12^, ^20^ As EC synthesis and insulin secretion are controlled by membrane depolarization and Ca^2+^ mobilization,^10^, ^11^, ^12^, ^15^, ^20^, ^40^ this supports the notion that the metabolically derived stimuli to insulin secretion also lead to EC generation and therefore should mean that insulin secretion and EC generation are proportional to one another. We have now found that ECs inhibit AC activity and that they inhibit cAMP accumulation in β cells, which results in diminished insulin secretion. Therefore, it seems reasonable to conclude that ECs limit insulin secretion under physiological conditions.

Activated CB1Rs are coupled to Gαi class of heterotrimeric G proteins and can initiate signalling events including closure of Ca^2+^ channels, opening of K^+^ channels and inhibition of AC activity (with its consequent decrease in cytosolic cAMP concentrations), resulting in inhibition of neurotransmitter release.^40^ However, the effects of CB1Rs on insulin release from β cells have not been firmly established, and the available data are occasionally inconsistent. We found that synthetic and endogenous ligands of CB1Rs reduced cAMP accumulation and insulin secretion in β cells and the CB1R inverse agonist prevented such effects. Moreover, Ex‐4‐ and forskolin‐mediated intracellular accumulation of cAMP was reduced by CB1R agonism in CB1R‐transfected CHO‐GLP‐1R cells, but not in vector‐transfected cells that intrinsically lack CB1Rs, further eliminating the possibility of any non‐specific effects of CB1R ligands. This is consistent with recent reports that show decreased intracellular cAMP levels in isolated islets 16 and MIN6 cells 28 due to ACEA. Taken together, we conclude that ECs influence insulin secretion in an autocrine manner by acting as a brake on AC activity.

Islets from *CB1R* ^−/−^ mice displayed increased intra‐islet insulin, in conjunction with insulin gene expression. Additionally, islets in pancreata from overnight‐fasted *CB1R* ^−/−^ mice displayed increased GCK and GLUT2 expression, compared to those from *CB1R* ^+/+^ mice. Moreover, knockout of IR abolished the ability of CB1R to regulate the expression of insulin, GCK and GLUT2, indicating that CB1Rs regulate their expression in IR signalling‐dependent manner. All of these factors combined are likely to be responsible for improved insulin secretion by CB1R antagonism. The molecular basis of the defective insulin secretion involves defects in the signal transduction pathways, especially such as insulin receptor signalling pathway, that are activated by increased glucose in pancreatic β‐cells.^41^, ^42^, ^43^ β‐cell dysfunction due to defective insulin receptor signalling leads to alterations in mechanisms that are responsible for the production and secretion of insulin, and this alteration involves a reduction of GLUT2 and GCK expression.^44^ βIRKO mice showed defective GSIS, progressive glucose intolerance, as well as reduction in GCK and GLUT2 gene expression in the islets of both non‐diabetic and diabetic βIRKO mice.^44^ In addition, islets from diabetic Zucker rats showed impaired GSIS associated with a reduction in the expressions of GLUT2 and GCK,^7^ and mice with a β‐cell–specific deletion of glucose‐sensing machinery revealed severe hyperglycaemia and infant death due to impaired GSIS. However, re‐expression of GLUT2 in β cells rescued the mice from infant death and restored normal GSIS,^8^, ^9^ showing that altered glucose‐sensing machinery of β cells leads to changes in GSIS. Taken together, it is possible to hypothesize that blockade of CB1R increases insulin secretion and improves insulin responsiveness, at least in part, by up‐regulation of insulin, GCK and GLUT2 gene expressions.

EC levels in circulating blood as well as the pancreas are reported to be elevated in diabetes and obesity,^10^, ^12^, ^45^, ^46^ but it is highly unlikely that islet‐derived ECs contribute to blood levels as they degrade within the islets. However, it is possible that increased EC tone (due to increased EC synthesis, receptor expression or activity) affects the well‐described incretin and glucose unresponsiveness of β cells in type 2 diabetes. Additionally, it was recently found that AEA impaired insulin‐stimulated AKT phosphorylation and decreased glucose uptake in skeletal muscle cells,^47^ while CB1R antagonism enhanced insulin responsiveness in skeletal muscle.^48^ Therefore, CB1R antagonists that have poor brain penetrance (to lessen CNS side effects) might be useful as therapeutic agents in type 2 diabetes where they would be expected to improve β‐cell function, improve glucose uptake into muscle and prevent or reduce hepatic steatosis.

## CONFLICT OF INTEREST

The authors confirm that there are no conflict of interests.

## AUTHOR CONTRIBUTIONS

S.H. and J.H.H. designed and performed experiments, analysed the data, and wrote the manuscript. H.J.S. performed the experiments. T.J.P. and E.K.L. analysed the data, provided reagents and reviewed the manuscript. JME designed the experiments, provided reagents, analysed the data and reviewed the manuscript. WK designed and performed the experiments and wrote, reviewed and edited the manuscript.
