# Supplementary material for: Blockade of cannabinoid 1 receptor improves glucose responsiveness in pancreatic beta cells
Source: J Cell Mol Med. 2018 Feb 12;22(4):2337–45. doi: 10.1111/jcmm.13523 (PMC5867156; doi:10.1111/jcmm.13523)
Supplement: Supplementary file 1 [file JCMM-22-2337-s001.docx]

| **Gene** | **Origin** | **Forward primer** | **Reverse primer** |
| --- | --- | --- | --- |
| *CB1R* | Mouse | 5’-GTGTTCCACCGCAAAGATAGT-3’ | 5’-GCCTGTGAATGGATATGTACCTG-3’ |
| *Gck* | Mouse | 5’-TGAGCCGGATGCAGAAGGA-3’ | 5’-GCAACATCTTTACACTGGCCT-3’ |
| *Glut2* | Mouse | 5’-CACATTCAAACTGACTTTCTGTTACC-3’ | 5’-TGTACGCAAAACCCGAAGTCT-3’ |
| *Ins1* | Mouse | 5’-TCTTCTACACACCCAAGTCCCG-3’ | 5’-CTCCAACGCCAAGGTCTGAA-3’ |
| *Ins2* | Mouse | 5’-GCTTCTTCTACACACCCATGTC-3’ | 5’-AGCACTGATCTACAATGCCAC-3’ |

**Table S1.** Primer Sequences Used in This Study
